# Corneal Crosslinking for Keratoconus in Iranian Patients: Outcomes at 1 year following treatment

**DOI:** 10.4103/0974-9233.71600

**Published:** 2010

**Authors:** Ladan Saffarian, Hamid Khakshoor, Mehran Zarei-Ghanavati, Habibollah Esmaily

**Affiliations:** Islamic Azad University of Mashhad, Mashhad, Iran; 1 Eye Research Center of Mashhad University of Medical Sciences, Khatam-al-Anbia eye Hospital, Mashhad, Iran; 2 Faculty of Health, Mashhad University of Medical Sciences, Mashhad, Iran

**Keywords:** Cornea, Crosslinking, Progressive Keratoconus, Riboflavin, Ultraviolet A

## Abstract

**Aim and Design:**

A retrospective, nonrandomized, single-center clinical study was designed to evaluate the outcomes of corneal collagen crosslinking (CXL) for progressive keratoconus in Iranian patients 12 months after CXL.

**Settings:**

This study was carried out at Navid Didegan Eye Center, a private clinic, Mashhad, Iran.

**Materials and Methods:**

Ninety-two eyes of 53 subjects with progressive keratoconus were evaluated in this study. All eyes completed 1-year follow-up. The outcome measures were uncorrected visual acuity (UCVA), best spectacle-corrected visual acuity (BSCVA), sphere and cylinder refraction, keratometry, and corneal thickness. Comparison of baseline and 1-year postoperative data is reported in this study. The Wilcoxon signed-ranked and Student’s *t*-tests were used for statistical analyses. *P* < 0.05 was statistically significant.

**Results:**

The mean age was 21.5 ± 3.4 years (range, 16 -30 years). Thirty-one (58.5%) of the subjects were men and 22 (41.5%) were women. Mean baseline UCVA and BSCVA were 0.61 ± 0.31 and 0.06 ± 0.12 logarithm of minimal angle of resolution (logMAR), respectively. One year postoperatively UCVA was 0.31 ± 0.25 logMAR and BSCVA was 0.0 ± 0.01 logMAR. UCVA and BSCVA were statistically higher postoperatively (*P* < 0.001, both parameters). The mean astigmatism decreased by 0.78 ± 1.49 diopter (D) with significant variation during the follow-up period (*P* < 0.001). Mean baseline simulated keratometry (SIM K) was 46.94 ± 2.37 D and decreased to 46.0 ± 2.33 D on year postoperatively (*P* < 0.001).

**Conclusion:**

Corneal CXL seems to be efficient in stabilization of progressive keratoconus progression in Iranian patients at 1 year of followup.

## INTRODUCTION

Keratoconus, a non-inflammatory disease, is characterized by progressive corneal thinning that results in a conelike ectasia, irregular astigmatism, and decreased vision. The incidence of keratoconus is reported to be 1 in 2000 in the general population.^1^,^2^ The onset is typically during puberty with slow progression.^3^ The condition often presents unilaterally yet does ultimately affect both eyes.^3^ Penetrating keratoplasty seems an essential surgical intervention in 20% of cases due to continued ectatic progression.^1^

Corneal crosslinking (CXL) treatment of keratoconus was first introduced by Wollensak *et al*. in 2003.^4^ CXL has an effect on the biomechanical properties of corneal collagen.^5^ Riboflavin, administered topically to de-epithelialized corneas, serves as a photosensitizer that is activated by UVA light. The light-induced production of oxygen radicals leads to the development of strong chemical bonds between collagen fibrils. With the aid of this new approach, stromal fiber photopolymerization enhances the corneal integrity and mechanical strength.^6^ Therefore, it may be considered as halting keratectasia progression during the progressive phase of keratoconus. In contrast, modal therapies such as rigid contact lenses, intracorneal rings, photorefractive keratectomy, or epikeratoplasty can be used only to correct the refractive errors of disease rather than to stop the keratoconus progression.^7^–^10^

The aim of this study was to evaluate outcomes of CXL in Iranian patients with progressive keratoconus, at 12 months after treatment.

## MATERIALS AND METHODS

### Cohort description and study criteria

In this retrospective, nonrandomized study, the cohort comprised 92 eyes of 53 subjects with progressive keratoconus. All procedures were performed at the private clinic, Navid Didegan Eye Center in Iran by a single surgeon. Informed consent was obtained from all subjects.

Inclusion criteria in this study were keratoconus progression and corneal thickness of ≥400 *µ*m at the thinnest point. Our criteria for keratoconus progression were exactly the same as a previous study:^11^ an increase in maximum keratometry (K) of 1.00 diopter (D) in 1 year; subject reports of deteriorating best corrected visual acuity (BCVA); or the need for new contact lens fitting more than once in 2 years.

Exclusion criteria were central or paracentral scars either in the epithelium or in the corneal stroma, corneal thickness less than 400 *µ*m, collagen vessel diseases, active ophthalmic inflammation, a history of herpetic keratitis, current corneal infection, pregnancy and lactation, and severe dry eye.

### Preoperative examinations

Examinations included measurement of uncorrected visual acuity (UCVA) and best spectacle-corrected visual acuity (BSCVA) by the Early Treatment Diabetic Retinopathy Study visual acuity chart (ETDRS) with a logarithm of minimal angle of resolution (logMAR) scale, dry, and cycloplegic refraction (after instillation of cyclopentolate 1%), slitlamp and funduscopic examinations, intraocular pressure with Goldmann applanation tonometry. Corneal topographic and pachymetry values were derived from Orbscan II (Bausch and Lomb, Rochester, NY, USA). Keratometry values reported in this study are the simulated keratometric values obtained with the Orbscan II.

### Surgical procedure

After topical anesthesia (0.5% tetracaine), mechanical epithelium removal of the central 8 mm area of the cornea was performed with a surgical blade (≠15) under sterile conditions. Prior to UVA irradiation, riboflavin 0.1% solution (10 mg riboflavin in 10 mL dextran 20% solution) was instilled every 2 min for 24 min. Penetration of adequate riboflavin was confirmed by slitlamp examination with a blue filter.

A UV-X device (IROC, Zürich, Switzerland) was used to deliver UVA irradiation. The instrument was set at a safe working distance of 5 cm from the corneal surface with medium size UV rays aperture at the wavelength of 370 nm and a surface irradiance of 3 mW/cm^2^. During the surgical procedure, riboflavin solution was applied every 4 min. A calibrated UVA meter (LaserMate-Q, LASER 2000, Wessling, Germany) was used to control the desired levels of irradiance before each treatment.

After treatment, the patient received topical ciprofloxacin 0.30% and betamethasone 0.1% with an extended-wear night and day bandage contact lens (CIBA Vision, Duluth, GA, USA) until complete re-epithelialization of the cornea.

### Postoperative management

Postoperatively, all patients were given topical ciprofloxacin and betamethasone (four times a day) for the first week. Subsequently, betamethasone was replaced with fluorometholone given four times daily for 1 week and then tapered by one drop every week over 1 month until cessation.

Postoperative examinations were carried out at one day and 7 days and 3, 6, and 12 months after surgery. The postexaminations were the same as the preoperative examinations.

### Statistical analysis

The Wilcoxon signed-ranked and Students *t*-tests were used for statistical analyses with SPSS software (SPSS, Inc., Chicago, IL, USA). Results as presented as mean values ± standard deviation. The threshold for statistical significance was set at a *P*-value less than 0.05. Preoperative and 1-year postoperative data are reported here.

## RESULTS

All patients completed 12 months postoperative follow-up. The mean age of the cohort was 21.5 ± 3.4 years (range, from 16-30 years). Thirty-one (58.5%) subjects were men and 22 (41.5%) were women.

### Visual outcome

**Figure 1:**
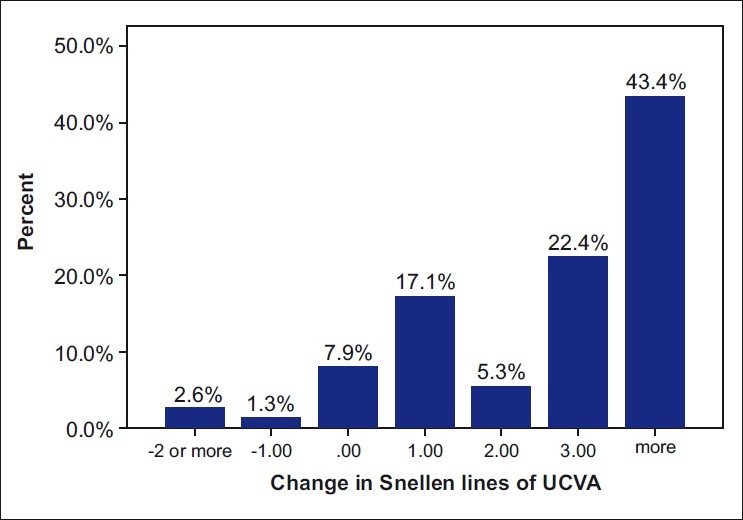
Change in uncorrected visual acuity (UCVA) 12 months after corneal crosslinking with riboflavin for progressive keratoconus

The improvement in UCVA was observed in 88.2% of eyes. Fifty eyes (54.3%) gained three or more lines of UCVA and two eyes (2.1%) lost two lines. The mean UCVA significantly improved 12 months after surgery from 0.61 ± 0.31 logMAR to 0.31 ± 0.25 logMAR (*P* < 0.001) [Figure 1]. The mean BSCVA increased significantly from 0.06 ± 0.12 preoperatively to 0.0 ± 0.01 logMAR postoperatively and after 1 year (*P* < 0.001). Eight eyes (8.6%) gained three or more lines of BSCVA.

Patients were categorized into two groups: eyes with three lines or more of UCVA gain (Group 1) and eyes with no change or loss of UCVA (Group 2). The following preoperative parameters of the two groups were compared using independent *t*-test: age; sphere; cylinder; spherical equivalent (SE); mean keratometry; corneal pachymetry; UCVA; and BSCVA. Group 1 had higher astigmatism (mean = 4.29 ± 1.55) and lower UCVA (mean = 0.74 ± 0.22) compared to Group 2 (mean = 3.27 ±0.79 and mean = 0.36 ±0.30; respectively) (*P* < 0.05). There were no statistically significant differences between the two groups for other parameters (*P* > 0.05, all parameters).

### Refractive outcome

The mean preoperative sphere was −1.06 ± 1.92 D, the mean cylinder was −3.93 ± 1.67 D, and the mean SE was −3.01 ± 2.05 D. Postoperatively, the mean sphere was −0.87 ± 1.60 D, the mean cylinder was −3.14 ± 1.50 D, and SE was −2.43 ± 1.79 D. A mean decline of −0.18 ± 0.79 D was observed in spherical power (*P* > 0.05). There was a statistically significant decrease in cylindrical power of 0.78 ±1.49 D (*P* < 0.001). SE also demonstrated a mean reduction of 0.57 ± 1.04 D(*P* < 0.001).

### Keratometric outcome

The mean keratometry was 46.94 ± 2.37 D before CXL and decreased to 46.0 ± 2.33 D 1 year postoperatively. A significant decrease of 0.94 ± 0.71 D was noted 1 year postoperatively (*P* < 0.001).

### Pachymetric outcome

Mean baseline corneal pachymetry was 460.68 ± 46.59 *µ*m (range, 400 to 576 *µ*m). One year postoperatively, the mean corneal thickness decreased significantly to 445.07 ± 41.57 *µ*m (range, 395 to 570 *µ*m) (*P* < 0.05).

### Complications

No significant complications such as persistent epithelial defect, infectious keratitis, or corneal haze and cataract formation occurred during this study.

## DISCUSSION

Interest in corneal CXL and the number of centers offering this new treatment continue to rise day after day. Despite this fact, long-term clinical studies are rare that evaluate safety and efficacy of CXL among different ethnicities. Treatment response may be different among racial and ethnic groups. Hence, it is necessary to obtain a data studies such as ours prior to considering CXL a standard treatment for keratoconus.

Our study like other previous studies shows that CXL with riboflavin is effective in halting the progression of keratoconus.^12^,^13^ For example, there was a mean decrease of 1 D in keratometric values in 90.5% of eyes in our study. Animal studies with quantitative biomechanical stress strain measurements have reported a significant increase in corneal rigidity porcine and rabbit corneas.^14^ Studies on humans with keratoconus have demonstrated satisfactory short-term and long-term findings with substantial topographic and refractive improvement following treatment.^15^ A considerable decline in topographic parameters such as keratometry, apical gradient curvature, inferior-superior index, cone area, and corneal aberrations after CXL has been reported.^16^

Similarly, we found an increase in UCVA and BSCVA was concomitant with topographic flattening and a decline in mean keratometry on year after CXL. Additionally, we found that patients with greater preoperative astigmatism and lower UCVA achieved a greater increase in UCVA after CXL. This outcome is consistent with Vinciguerra *et al*.,^16^ who reported a statistically significant decrease in cylinder and improvement in UCVA and BSCVA after CXL in eyes with progressive keratoconus. Caporossi *et al*.^15^ illustrated that the mean SE declined 2.5 D, postoperatively. These results were topographically confirmed by a reduction in mean keratometry. Corneal aberrometry indicated improvements in corneal morphologic symmetry.^15^

No significant adverse effects were observed among the subjects in our study. In our study, all subjects had corneal thickness greater than 400 *µ*m. Subjects with corneal pachymetry less than 400 *µ*m were excluded on the basis of Wollensak *et al*.’s report that a standard surface UVA dose of 3 mW/cm has a toxic effect on endothelial cells of corneas thinner than 400 *µ*m.^17^

CXL is a minimally invasive procedure. This novel method can halt or reduce keratoconus progression in comparison to other treatment approaches which only have a transient effect on refractive corrections. Therefore, CXL seems to be the primary approach to stop or decrease the progression of keratoconus. As keratoconus is prevalent in Iran and it is the most common cause of corneal transplantation in our country,^18^ we believe that CXL can play a key role in reducing the necessity of corneal transplantation in keratoconus patients. Furthermore, there is added advantage of obviating the risks and complications associated with keratoplasty. The simplicity and minimal invasiveness of this procedure can precipitate a significant shift in treatment of progressive keratoconus in countries where the approach to corneal transplantation has various difficulties.

In conclusion, our study not only demonstrated a halt in keratoconus progression, but also showed a significant improvement in visual acuity and refractive results due to a decline in the irregular astigmatism after CXL. These outcomes provide an indication of the efficacy and safety of this new approach and the potential for CXL to be an appropriate therapy for progressive keratoconus among Iranian patients. However, further trials with longer follow-up are essential to confirm these encouraging results.
